# Cocaine locomotor activation, sensitization and place preference in six inbred strains of mice

**DOI:** 10.1186/1744-9081-7-29

**Published:** 2011-08-01

**Authors:** Amy F Eisener-Dorman, Laura Grabowski-Boase, Lisa M Tarantino

**Affiliations:** 1 Department of Psychiatry, University of North Carolina, Chapel Hill, NC, USA; 2 Genomics Institute of the Novartis Research Foundation, San Diego, CA, USA

## Abstract

**Background:**

The expanding set of genomics tools available for inbred mouse strains has renewed interest in phenotyping larger sets of strains. The present study aims to explore phenotypic variability among six commonly-used inbred mouse strains to both the rewarding and locomotor stimulating effects of cocaine in a place conditioning task, including several strains or substrains that have not yet been characterized for some or all of these behaviors.

**Methods:**

C57BL/6J (B6), BALB/cJ (BALB), C3H/HeJ (C3H), DBA/2J (D2), FVB/NJ (FVB) and 129S1/SvImJ (129) mice were tested for conditioned place preference to 20 mg/kg cocaine.

**Results:**

Place preference was observed in most strains with the exception of D2 and 129. All strains showed a marked increase in locomotor activity in response to cocaine. In BALB mice, however, locomotor activation was context-dependent. Locomotor sensitization to repeated exposure to cocaine was most significant in 129 and D2 mice but was absent in FVB mice.

**Conclusions:**

Genetic correlations suggest that no significant correlation between conditioned place preference, acute locomotor activation, and locomotor sensitization exists among these strains indicating that separate mechanisms underlie the psychomotor and rewarding effects of cocaine.

## Background

The devastating effects of drug addiction on the lives of those who struggle with it and the social and economic implications for society as a whole are staggering. Faced with this challenge, understanding the biological pathways that predispose individuals to addiction to cocaine (and other drugs) is a top priority in the research community. The perception that genetic background influences the predisposition to abuse drugs is supported by studies in humans [1] and in animal models [2-4]. While no animal model exists that recapitulates the entire spectrum of the drug abuse syndrome in humans, animal models do exist for the study of specific drug-related behaviors, including initial sensitivity (as measured by drug-induced locomotor behavior), sensitization and drug reward and reinforcement paradigms.

Conditioned place preference (CPP) has been established as a standard procedure for assessing the rewarding effects of drugs in rodent models. In the CPP paradigm, rodents learn to associate a specific environmental context with the effects of a drug stimulus. Place conditioning confers many advantages in the study of drug-induced motivational responses, including the short duration and relative simplicity of the procedure in comparison with reinforcement models such as self-administration. CPP also provides the opportunity to measure the initial acute response and sensitization or tolerance to the locomotor stimulating effects of a drug following repeated exposure [5,6]. In addition, CPP is measured in the absence of drug-related physiological confounds such as locomotor and sensory effects [5]. Encountering environments and stimuli previously associated with drug use is one of the most common triggers for relapse in humans [7,8], and the CPP paradigm specifically addresses the effects of context-specific exposures on drug reward and drug-seeking behaviors in animal models [9].

Drug-induced behaviors, including CPP, have a significant genetic component as demonstrated by the large degree of phenotypic variability among inbred mouse strains [4,10]. The goal of the present study was to expand upon the current literature by investigating drug-induced sensitivity, sensitization and place conditioning in six commonly used and genetically diverse inbred mouse strains. Several of these strains have already been characterized for CPP, cocaine locomotor activation and sensitization [4], while others, including FVB/NJ and substrains of 129 (129S1/SvImJ) and BALB (BALB/cJ), have not previously been studied in the place preference paradigm. Here, we present strain-specific differences that confirm, contradict, and expand upon previously reported inbred strain responses to cocaine.

## Methods

### Animals

Male mice from six inbred strains (*129S1/SvImJ (129), C57BL/6J (B6), BALB/cJ (BALB), C3H/HeJ (C3H)*, *DBA/2J (D2) *and *FVB/NJ (FVB)*) were obtained from the in-house breeding colony at the Genomics Institute of the Novartis Research Foundation (GNF). Inbred strains for the GNF breeding colony were initially purchased from the Jackson Laboratory (Bar Harbor, ME, USA), and breeder stocks were replenished every seventh generation to limit genetic drift. Mice were group-housed and maintained in an AAALAC-accredited, specific pathogen-free (SPF) barrier colony in ventilated cages (Thoren Caging Systems, Hazelton, PA, USA) on a 12-hour light-dark cycle (lights on at 6:00 A.M.). Irradiated food (Pico rodent chow 20; Purina, St. Louis, MO, USA) and water were provided *ad libitum*. Mice were between 59 and 70 days of age at the onset of testing, and all behavioral testing occurred between 8:00 A.M. and 12:00 P.M.

Sixteen B6 mice and eight mice from each of the remaining five strains were tested in the conditioned place preference procedure described below. Groups of mouse strains were tested in three separate testing sessions over the course of two months, and all mice in a group were tested on the same day. With the exception of B6, all mice of each strain were tested in the same session and different strains were tested in each of the three sessions. Two sets of B6 mice (8 mice per set) were tested in the first and last sessions as a control for temporal effects across sessions.

Male mice were chosen for testing to mitigate the variability observed in females due to estrous cycle [11-13].

All procedures were approved by the GNF Institutional Animal Care and Use Committee and followed the guidelines set forth by the National Institutes of Health (NIH) Guide for the Care and Use of Laboratory Animals.

### Drugs

Cocaine was dissolved in saline (0.9% NaCl). A 20 mg/kg dose of cocaine was used to obtain a moderate cocaine-induced response across strains, as previous studies suggest this dose is sufficient to induce a drug response in many strains [4,14,15]. Immediately following an intraperitoneal (i.p.) injection with cocaine or saline, each animal was placed in the appropriate chamber of the CPP apparatus.

### Apparatus

Conditioned place preference testing was conducted using a three-chambered conditioned place preference apparatus (46.5 × 12.7 × 12.7 cm; MED-CPP-MSAT, Med Associates) in a sound-attenuating enclosure (ENV-016MD, Med Associates). The apparatus consisted of a grey center compartment (7.2 × 12.7 cm) with a smooth PVC floor and two choice compartments (16.8 × 12.7 cm) on either side. One compartment was all black with a stainless steel grid rod floor consisting of 3.2 mm rods placed on 7.9 mm centers and the other was all white with a 6.35 × 6.35 mm stainless steel mesh floor. Compartments were separated by guillotine doors, which were left open to permit free exploration of the full apparatus during pre-test on Day 1 and the testing phase on Day 10 but were closed during training days. Two identical CPP apparatuses were used and each apparatus had two training chambers (black and white) for a total of four training chambers. Each mouse was tested in the same apparatus and the same training chamber throughout the experiment.

Mouse movement in the apparatus was detected by six infrared photobeams (spaced 2.8 cm apart and 1.0 cm from the end wall) in each choice compartment and three (spaced 2.8 cm apart) in the center compartment. Photobeams mounted 1.8 cm above the apparatus floor captured horizontal movement. The absence of z-axis photobeams did not permit detection of vertical movement, such as rearing. Activity was captured by automated data collection as movement counts. Movement counts are defined as consecutive beam breaks within a chamber to detect horizontal forward locomotion while excluding stereotypic behavior. Entrance into a chamber was recorded when the second photobeam into the chamber was broken. Time spent in each compartment was recorded in seconds.

### Conditioned place preference procedure

**Figure 1:**
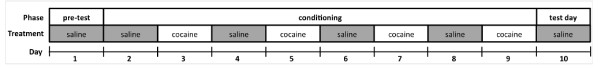
**Timeline of the place preference study**.

The place preference procedure consisted of three phases, as outlined below and in Figure 1.

#### Pre-test phase

The purpose of the pre-test was to habituate the animals to the novelty and stress associated with the apparatus, handling and injection prior to conditioning, as well as to identify initial chamber preferences. For the pre-test, all mice received an i.p. saline injection immediately prior to placement in the apparatus for 20 minutes. Mice were placed in the grey center compartment at the beginning of the pre-test but were allowed to move freely between compartments. The total time spent in each of the three chambers was recorded and then calculated as a percentage of the total test duration. The percent time spent in the black and white chambers only, not including the grey chamber, was also calculated and used as the pre-conditioning value for place preference analysis.

#### Conditioning phase

For the conditioning procedure, animals from each strain were randomly assigned to one of two conditioning subgroups. Mice in one subgroup (N = 4 per strain; B6 N = 8) received cocaine paired with the black chamber and saline paired with the white chamber. Mice in the second subgroup (N = 4 per strain; B6 N = 8) received cocaine paired with the white chamber and saline paired with the black chamber. On days 2, 4, 6 and 8, mice were injected with saline and placed in the unpaired chamber. On days 3, 5, 7 and 9, mice were injected with cocaine and placed in the drug-paired chamber. Conditioning took place across eight subsequent days, and trials were 30 minutes in duration (one trial per day). Movement counts in the black and white chambers were recorded.

#### Test day

For the testing phase on Day 10, mice received an i.p. injection of saline immediately prior to placement in the grey center compartment of the apparatus and were allowed to move freely between the compartments for the duration of the 30-minute testing trial. The primary dependent variable was percent time spent in the cocaine-paired chamber during the testing trial, which was calculated by dividing time spent in the drug-paired chamber by total time spent in the black and white chambers and multiplying by 100. Conditioned place preference was measured as percent time spent in the cocaine-paired chamber before (Day 1) and after (Day 10) conditioning, and the development of place preference was defined as a significant difference between pre- and post-conditioning values. Movement counts in the black and white compartments were also recorded.

### Statistical analysis

All data were analyzed using the SPSS statistical package (version 16.0 for Macintosh, SPSS, Chicago IL, USA). Multivariate analysis of variance (ANOVA) was used to analyze the effect of strain for each behavioral measure. Dependent variables differed based on behavioral measure and included percent time spent in each chamber for equipment bias assessment and percent time spent in the cocaine-paired chamber for place preference. Locomotor activity (recorded as movement counts) was assessed for acute locomotor response, sensitization and on the test day (Day 10). Day of testing was included as an independent variable to assess the effects of cocaine on time spent in the cocaine-paired chamber and also for locomotor activity over multiple days of testing (acute and sensitized locomotor activity). Based on results from the pre-test session indicating that the equipment was unbiased, data were collapsed across training chambers for analysis of place preference. However, locomotor activity during the conditioning phase differed between black and white chambers for some strains; therefore, chamber was included as an independent variable for activity analyses. Locomotor activity was also used as a covariate in the CPP analysis. *Post-hoc *Tukey HSD and t-tests were employed for individual *post-hoc *comparisons.

The two groups of B6 mice were analyzed by one-way ANOVA with group as the independent variable. Dependent variables included acute locomotor response, sensitization and place preference.

Genetic correlations were assessed using partial correlation of percent CPP (Day 10 - Day 1), total locomotor activity in the black and white chambers on the test day (Day 10 movements), cocaine locomotor sensitization (Day 9 - Day 3 movements), acute locomotor response to cocaine (Day 3 - Day 2 movements) and saline-induced basal locomotor activity (Day 2 movements), therefore controlling for the effects of strain. Percent time spent in the drug-paired chamber on Day 1 was subtracted from percent time spent in the drug paired chamber on Day 10 to yield the CPP value used for correlation analyses.

## Results

### Pre-test chamber bias

**Figure 2:**
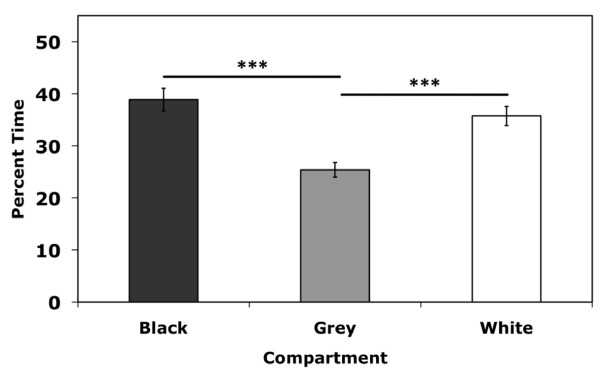
**Initial bias for each chamber of the three-chamber place preference apparatus during the pre-test session**. All strains were included in the analysis. Separate analysis excluding 129 mice yielded similar results (data not shown). Error bars are SEM. ***p < 0.001.

Training chamber bias was examined by two-way ANOVA (strain and chamber) of time spent in each of the three chambers. A significant main effect of chamber was observed (F_(2,167) _= 16.6;*p *< 0.001) as well as a strain by chamber interaction (F_(10,167) _= 4.5;*p *< 0.001). Tukey *post-hoc *comparisons of the three chambers indicated that there was no difference in the time mice spent in the black and white chambers (*p *= 0.374) (Figure 2). Therefore, mice were assigned to the training chamber in an unbiased manner and place preference data analysis was conducted on the group as a whole regardless of training chamber.

Visual inspection of Day 1 data indicated that three out of eight 129 mice spent almost 100% of the time in a single chamber during the pre-test, and one mouse spent over 83% (data not shown). Therefore, 129 mice were excluded from overall analysis of place preference to avoid skewing interstrain comparisons due to 129 strain-specific equipment bias effects. Place preference in the 129 mice was analyzed separately from the other strains with the four outliers excluded.

### B6 Control Groups

One-way ANOVA identified no significant effect of B6 group on either CPP (F_(1,15) _= 0.21;*p *> 0.05) or sensitization (F(1,15) = 0.13;*p *> 0.05). A significant effect of acute locomotor response to cocaine was observed (F(1,15) = 4.9;*p *< 0.05). Animals in Group 1 show greater acute sensitivity to cocaine than animals in Group 2. A closer examination of the data indicates that the groups do not differ on Day 2 baseline locomotor activity (t(14) = 0.26;*p *> 0.05), but B6 animals from Group 1 are significantly more activated by cocaine on Day 3 (t(14) = 2.4;*p *< 0.05). Extending the analysis to the entire experiment, we observed that the two groups do not differ for saline-induced locomotor behavior (Days 2, 4, 6 and 8) but consistently differ for cocaine-induced locomotor behavior (Days 3, 5, 7 and 9) (data not shown).

### Cocaine place preference

**Figure 3:**
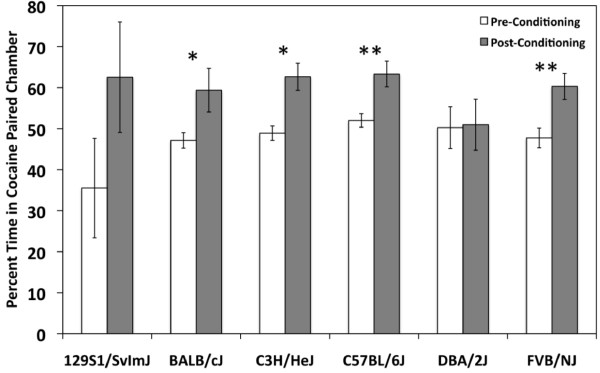
**Cocaine-induced place preference in six inbred strains**. Percent time spent in the cocaine-paired chamber both pre- (white bars) and post-conditioning (grey bars) is shown. Error bars are SEM. *p < 0.05, **p < 0.01.

A two-way ANCOVA (strain by day with locomotor activity as the covariate) yielded a significant effect of day (F_(1,95) _= 7.3;*p *< 0.01) indicating that mice spent more time in the cocaine-paired chamber on Day 10 following conditioning than on Day 1 during the pre-test. The effect of locomotor activity was not significant (F_(1,95) _= 0.78;*p *> 0.05). Strain (F_(4,95) _= 1.5;*p *> 0.05) and strain by day interaction effects (F_(4,95) _= 1.2;*p *> 0.05) were also not observed indicating that no strain differences in place preference were detected. *Post-hoc *t-tests, however, showed that D2 mice did not spend significantly more time in the cocaine-paired chamber on Day 10 than on Day 1 (t(14) = -0.09;*p *> 0.05; Figure 3).

129 mice spent more time in the drug-paired chamber after conditioning, but the difference was not significant (t(14) = -1.5;*p *> 0.05) indicating that this substrain of 129 does not exhibit place preference to 20 mg/kg under these experimental conditions (Figure 3). However, variability in 129 mice was much higher than in other strains, possibly due to locomotor hypoactivity often observed in this inbred strain.

### Chamber effects on locomotor activity

Differences in locomotor activity in the black and white chambers are an important consideration when analyzing locomotor response to cocaine in the CPP procedure. Although strains spent equal amounts of time in the black and white chambers on Day 1, two-way ANOVA (strain × chamber) of locomotor movements in the CPP apparatus during the pre-test indicated that there were significant main effects of strain (F_(5,111) _= 28.2;*p *< 0.001) and chamber (F_(1,111) _= 39.1;*p *< 0.001), as well as a significant interaction effect (F_(5,111) _= 2.5;*p *< 0.05). Strain differences were reflective of normal variation in locomotor activity among inbred strains and had the following pattern: 129 = C3H < B6 = D2 = FVB < BALB. Overall, mice were more active in the black chamber. *Post-hoc *t-tests indicated that increased activity in the black chamber was only significant for B6 (t(30) = 6.8;*p *< 0.001), D2 (t(14) = 3.9;*p *< 0.01) and FVB (t(14) = 5.5;*p *< 0.001) strains.

However, locomotor activity differences in black vs. white chambers were no longer significant on the second day of testing when mice were restricted to the unpaired chamber in which they received saline and before their first exposure to cocaine. On Day 2, only strain effects were significant (F_(5,55) _= 6.6;*p *< 0.001) and no chamber (F_(1,55) _= 0.75;*p *= 0.39) or interaction effects (F_(5,55) _= 1.3;*p *= 0.27) were observed. Nevertheless, cocaine-paired chamber was included as an independent variable in all locomotor activity analyses to assess the effect of chamber on the dependent variable.

### Acute locomotor response to cocaine

**Figure 4:**
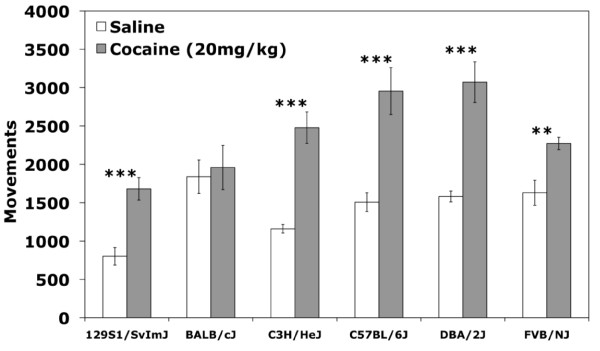
**Acute locomotor response across strains to a single 20 mg/kg cocaine challenge**. Saline locomotor activity (white bars) was recorded on day 2 of testing and cocaine locomotor activity (grey bars) was recorded on day 3. Error bars are SEM. **p < 0.01, ***p < 0.001.

Acute locomotor response was measured by comparison of locomotor activity on Day 2 (saline) and Day 3 after the first exposure to cocaine. Significant strain (F_(5,111) _= 6.46;*p *< 0.001) and day of testing (F_(1,111) _= 62.1;*p *< 0.001) effects and a significant strain by day of testing interaction effect (F_(5,111) _= 3.23;*p *< 0.05) were observed by two-way ANOVA. *Post-hoc *t-tests of individual strains indicate that all strains showed a significant increase in locomotor activity in response to acute cocaine with the exception of BALB (Figure 4). No effect of cocaine-paired chamber was observed (F_(1,111) _= 0.19;*p *= 0.66) indicating that acute locomotor activation was not affected by the chamber in which the mice received cocaine.

### Cocaine locomotor sensitization

**Figure 5:**
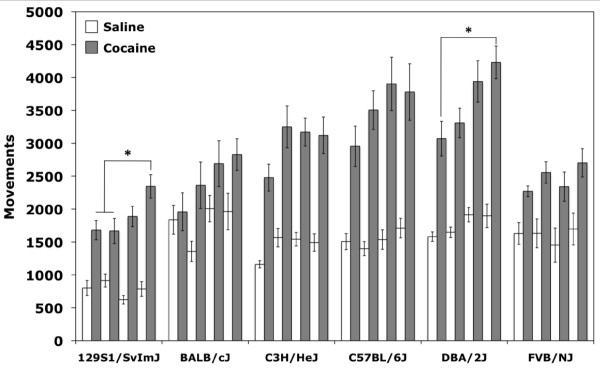
**Cocaine locomotor sensitization across strains**. Locomotor activity for 30-minute sessions across eight days of conditioning. Saline treatment days (white bars) and cocaine treatment days (grey bars) are shown. Error bars are SEM. *p < 0.05.

Locomotor sensitization for all strains was assessed by examining locomotor behavior across all four days of cocaine treatment. Significant main effects of strain (F_(5,223) _= 18.2;*p *< 0.001) and day of treatment (F_(3,223) _= 5.9;*p *< 0.01) but no strain by day interaction effect (F_(15,223) _= 0.42;*p *= 0.97) were observed by ANOVA. Cocaine-induced locomotor activation increased with repeated treatments across all strains indicating that sensitization was occurring. *Post-hoc *Tukey comparisons collapsed across strains indicate that locomotor behavior increased significantly by the third cocaine challenge. However, although most strains showed a pattern of increasing locomotor response with repeated exposures, *post-hoc *Tukey's analysis by strain indicates that only 129 and D2 mice showed significant increases in locomotor activity after the initial dose of cocaine **(**Figure 5**)**.

Locomotor activity in response to saline was also assessed by two-way ANOVA, and a main effect of strain was observed (F_(5,223) _= 18.7;*p *< 0.001) but no day of treatment (F_(3,223) _= 1.5;*p *= 0.206) or strain by day interaction effects (F_(15,223) _= 1.1;*p *= 0.380). However, no generalized locomotor changes across days of saline treatment were observed (Figure 5).

**Figure 6:**
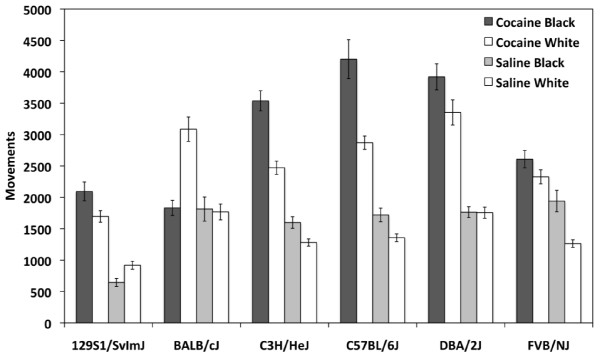
**Effect of training chamber on locomotor activity during repeated exposures to either cocaine or saline**. Bars represent locomotor activity of groups of mice (N = 4 per strain; B6 N = 8) exposed to cocaine and saline in either the black or white chamber. The first white bar is cocaine-induced activity in white chambers. The second white bar is saline-induced activity in white chambers. Error bars are SEM. B6, C3H and FVB exhibited more locomotor activity in the black chamber regardless of treatment with either saline or cocaine. D2 mice showed no difference in activity in either black or white chambers. 129 mice showed reciprocal differences in activity and were more active in the black chamber after exposure to cocaine and more active in the white chamber after exposure to saline. BALB mice showed similar amounts of locomotor activity in response to saline in both the black and white chambers and showed a significantly higher response to cocaine only when it was administered in the white chamber.

An effect of cocaine-paired chamber was also observed during both cocaine treatment days (F_(1,223) _= 10.9;*p *< 0.01) and saline treatment days (F_(1,223) _= 8.8;*p *< 0.01), as well as strain by cocaine-paired chamber interaction effects (F_(5,223) _= 10.2;*p *< 0.001 and F_(5,223) _= 4.3;*p *< 0.01, respectively). These data indicate that inbred strains differed in their locomotor behavior depending upon the chamber to which they were restricted during the conditioning trials. Several strains, including B6, C3H and FVB, appear to have a generalized increase in locomotor activity in the black chamber regardless of treatment with either saline or cocaine (Figure 6). D2 mice showed no difference in activity in either black or white chambers. Two strains in particular, BALB and 129, exhibited a chamber and/or drug-dependent locomotor response. 129 mice showed reciprocal differences in activity and were more active in the black chamber after exposure to cocaine and more active in the white chamber after exposure to saline. BALB mice showed similar amounts of locomotor activity in response to saline in both the black and white chambers and, surprisingly, showed a significantly higher response to cocaine only when it was administered in the white chamber (Figure 6).

### Correlation analyses

Partial correlations were performed to assess relationships between the stimulatory and rewarding effects of cocaine while controlling for strain effects. The effect of locomotor activity on the test day was assessed with regard to its effect on place preference for all strains except 129. A partial correlation of total locomotor movements on Day 10 and percent place preference indicated that activity did not have a significant effect on place preference behavior (Table 1; r(45) = 0.21;*p *> 0.05).

**Table 1:** Partial correlations of behavioral variables controlling for strain

		Acute Locomotor Stimulation	Locomotor Sensitization	Day 10 Movements	Baseline Locomotor Activity
**Locomotor Sensitization**	*r*	-0.077			
	*p*	0.607			
	*df*	45			

**Day 10 Movements**	*r*	0.004	0.153		
	*p*	0.976	0.303		
	*df*	45	45		

**Baseline Locomotor Activity**	*r*	*-0.402*	-0.035	*0.487*	
	*p*	*0.005*	0.815	*0.001*	
	*df*	*45*	45	*45*	

**Place Preference**	*r*	-0.057	0.055	0.214	-0.091
	*p*	0.704	0.711	0.149	0.543
	*df*	45	45	45	45

The relationships between acute locomotor stimulation, sensitization and CPP were also assessed by partial correlation and the results presented in Table 1. No significant correlations were observed.

A relationship between baseline and acute locomotor activity in response to cocaine has been previously reported [16,17]. Partial correlation analysis of saline-induced baseline activity and cocaine-induced locomotor activation did show a significant negative correlation (r(45) = -0.40; *p *< 0.01) indicating that strains with higher baseline locomotor activity exhibited lesser increases in locomotor response to cocaine.

## Discussion

In this study of six commonly-used inbred strains, our results show significant strain differences in locomotor response to an acute cocaine challenge, in addition to locomotor sensitization and conditioned place preference to cocaine for most strains. Despite the absence of CPP in 129S1/SvImJ and DBA/2J mice, these strains exhibited robust acute locomotor and sensitization responses to 20 mg/kg of cocaine. Both FVB/NJ and BALB/cJ developed CPP, but FVB showed low acute locomotor activation and no significant sensitization, whereas BALB showed no acute locomotor activation but strong context-specific cocaine sensitization. C57BL/6J and C3H/HeJ mice developed CPP as well as strong acute locomotor stimulatory and sensitization responses. The results of this study highlight the significant role of genetic background in determining behavioral responses to drugs of abuse, such as cocaine.

It should be noted that these results are limited to a single dose of 20 mg/kg and dose-dependent differences in place preference have been noted by others [18-20]. In addition, other behaviors such as rearing or stereotypies may interfere with the measurement of locomotor behavior. In general, peripherally-administered cocaine results in decreased rearing behavior, although this effect is strain- and dose-dependent [21,22]. Measurement of rearing behavior is not possible with our CPP apparatus and strain differences in rearing behavior could have affected locomotor response; however, at least one study has shown dissociation between cocaine's effects on rearing and locomotor behaviors [23]. Stereotypy in response to cocaine is also dose- and strain-dependent. Stereotypic responses to repeated, but not acute, injections of cocaine have been reported for B6 and D2 strains [24,25]. Strain differences in stereotypy can also interfere with locomotor responses and may play a factor in our results. Regardless, strain differences in locomotor activity due to stereotypy still reflect real differences in behavioral sensitivity to cocaine.

Finally, differences in cocaine-induced locomotor activation in the B6 groups suggest that the strain differences in locomotor response to cocaine could be confounded by temporal differences. However, we do not believe this to be the case based on the congruence of our strain data with published studies on cocaine locomotor activation.

Our study includes three strains or substrains that, to the best of our knowledge, have not yet been characterized for cocaine-induced CPP - FVB/NJ, 129S1/SvImJ and BALB/cJ. FVB mice and 129 ES cells are frequently used in the generation of transgenic and knockout lines, therefore, characterization of these strains' responses to the rewarding and stimulating properties of cocaine has significant implications for behavioral effects related to genetic background. Similar to the results of a study by Zombeck et al. [26], we found that acute locomotor response to 20 mg/kg cocaine in FVB mice is lower relative to the other strains, except BALB (Figure 4). FVB mice also do not show significant sensitization to repeated doses of cocaine under our experimental conditions (Figure 5). Although acute locomotor response to cocaine is relatively low, FVB mice do show robust place preference (Figure 3) indicating that this strain is sensitive to the rewarding effects of cocaine.

Cocaine-mediated behaviors in the 129S1 strain have not yet been reported; however, several other 129 substrains have been characterized for cocaine locomotor activity with mixed results [21,25,27-29]. Taken together, these results suggest that locomotor activation in 129 mice may be substrain or dose-dependent and may be sensitive to experimental parameters.

Studies assessing the rewarding effects of cocaine in 129 substrains are more variable with some studies observing no place preference [28] and others observing significant place preference [27]. We found that 129S1 mice do not show significant place preference to 20 mg/kg cocaine, similar to the observation of Miner [28] although at lower doses. Our results may have been influenced by the baseline hypoactivity observed in 129S1 mice, which are extremely inactive in many behavioral assays [30-32]. These data underscore the importance of considering the unique behavioral characteristics of different inbred mouse strains that may influence experimental outcomes.

As we observed in the 129 mice, the role of locomotor activity in behavioral assays cannot be overlooked. The extent to which basal locomotor activity correlates with initial drug sensitivity has been examined with varied results. Several studies report no correlation between acute locomotor stimulation and baseline locomotion [33,34], while others observe a significant correlation [16,17]. In our sample, baseline locomotor activity does predict acute response to cocaine, as demonstrated by the correlation between saline-induced and acute cocaine-induced activity.

Correlations between activity and CPP may reflect interference between conditioned activity and the development of place preference or strain differences in either basal or psychostimulant-induced locomotor response during training [35-37]. Conditioned activity does not appear to influence CPP in our group, as we did not observe a correlation between CPP and locomotor activity on the test day.

The measurement of acute response, sensitization and the rewarding effects of cocaine allow us to examine one prominent hypothesis of addiction. The incentive-sensitization model of drug craving suggests that chronic drug abuse causes hypersensitivity of the underlying brain circuitry as measured by sensitization, which exacerbates incentive salience and may explain the compulsive drug-seeking that drives addiction [38]. Similar to observations by Cunningham et al. [14], our data do not appear to support theories of a positive link between drug craving and sensitization [38] or the rewarding and stimulating effects of drugs [39], as we observed no correlation between place preference and either acute locomotor stimulation or sensitization.

**Figure 7:**
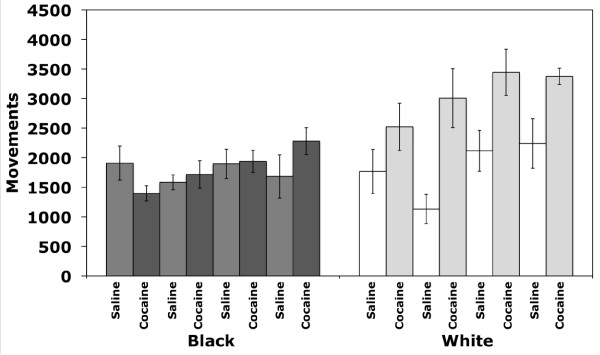
**White vs. black chamber effect on cocaine-induced locomotor activity and sensitization observed for BALB/cJ mice in the CPP apparatus**. Results are separated by chamber in which mice received cocaine during conditioning trials. Conditioning days and treatment are shown sequentially along the x-axis. Error bars are SEM.

Similar to our results, most studies in BALB mice report low levels of locomotor activation or no effect at all in response to cocaine [22,26,40-46]. Further, BALB mice reportedly do not develop place preference to moderate cocaine doses [18]. However, the level of locomotor activation in our study was dependent on the chamber in which the cocaine was administered. BALB mice receiving cocaine in the white chamber showed significant locomotor activation that exceeded the responses of other strains whereas BALB mice receiving cocaine in the black chamber did not display locomotor activation (Figure 6). This level of activation was not observed after acute administration of cocaine but was observed on all subsequent days of cocaine administration in the white chamber (Figure 7) indicating that the locomotor activation developed upon repeated drug dosing. Taken together, these data indicate that a specific interaction between the drug and the environment is occurring, resulting in context-dependent sensitization in BALB mice.

The context in which a drug is experienced can significantly influence both acute and sensitized responses to the drug [47,48]. Context-dependent sensitization has also been attributed to associative learning or increased stress [47,49-51]. It is possible that the white chamber may be anxiogenic for BALB mice, resulting in greater sensitization. However, this is unlikely since BALB mice spend equivalent amounts of time in both chambers during the pre-test session on Day 1. Alternatively, features of the white chamber may cause BALB mice to more readily associate the context with the drug effects. Willner et al. have shown that the extent of sensitization is determined by the behavior elicited by the drug [52], thus BALB mice may develop greater sensitization as a result of a greater stimulatory response to cocaine in the white chamber. Acute locomotor response to cocaine is certainly higher for BALB mice in the white chamber (Figure 7) but this does not explain why this strain, in particular, shows greater sensitivity to the psychomotor effects of cocaine in this context. The mechanisms by which drug effects interact with context to result in behavioral differences remain to be determined. Further experiments are necessary to replicate these results and to gain a better understanding of this phenomenon in BALB mice.

Our observation that D2 mice did not exhibit significant place preference under our assay conditions aligns with previous reports using higher doses of cocaine (32 mg/kg [4]; 30 mg/kg [14]) and deviates from others reporting place conditioning in response to moderate doses (10 mg/kg [14]). Taken together, these studies support dose-dependent place preference in D2 mice. However, modification of experimental parameters can also influence place conditioning and should be carefully considered when planning experiments and reporting results [14].

## Conclusions

This study expands upon current literature in the field, describing cocaine-induced conditioned place preference in three strains/substrains for which it had not previously been reported. Further, we have also shown context-dependent locomotor sensitization in response to cocaine in BALB/cJ mice. Of the strains included in our study, B6 and C3H emerge as the most appropriate for the study of responses to cocaine, as these mice develop place preference and show acute locomotor activation and sensitization. The use of strains such as FVB and BALB may benefit studies aiming to dissociate the mechanisms underlying these cocaine-induced behaviors. These results highlight the importance of considering both context and genetic background in the analysis of cocaine-induced behaviors.

Much remains unknown regarding the neural circuitry and genetic mechanisms underlying drug addiction. The tremendous genetic variability among inbred mouse strains and our ability to model aspects of addiction with various behavioral paradigms, such as CPP, will eventually lead to the identification of genes and gene networks that influence addiction pathways in the brain and promise to have a profound effect on the treatment of addiction in the clinic.

## Competing interests

The authors declare that they have no competing interests.

## Authors' contributions

AFED contributed to the statistical analysis and the drafting of the manuscript. LGB carried out the behavioral testing and contributed to the study design. LMT conceived of the study, participated in its design and coordination, contributed to the statistical analysis and helped to draft the manuscript. All authors read and approved the final manuscript.
